# α-Fe_2_O_3_/graphene oxide powder and thin film nanocomposites as peculiar photocatalysts for dye removal from wastewater

**DOI:** 10.1038/s41598-021-99849-x

**Published:** 2021-10-13

**Authors:** Mahsa Khoshnam, Javad Farahbakhsh, Masoumeh Zargar, Abdul Wahab Mohammad, Abdelbaki Benamor, Wei Lun Ang, Ebrahim Mahmoudi

**Affiliations:** 1 Material Science and Engineering Department, Isfahan University of Technology, Isfahan, Iran; 2 School of Engineering, Edith Cowan University, Joondalup, WA 6027 Australia; 3 Chemical Engineering Department, University Kebangsaan Malaysia, Bangi, Malaysia; 4 Centre for Sustainable Process Technology (CESPRO), Faculty of Engineering and Built Environment, Universiti Kebangsaan Malaysia, UKM, 43600 Bangi, Selangor Malaysia; 5 Gas Processing Centre, Qatar University, P.O. Box 2713, Doha, Qatar

**Keywords:** Climate sciences, Environmental sciences, Engineering, Materials science

## Abstract

In this study, hematite graphene oxide (αFe_2_O_3_-GO) powder nanocomposites and thin-film hematite graphene oxide (αFe_2_O_3_-GO) were synthesized for application in the removal of Rhodamine B (RhB) from textile wastewater. αFe_2_O_3_-GO nanomaterials were placed onto the FTO substrate to form a thin layer of nanocomposites. Different analysis including XRD, FTIR, Raman spectra, XPS, and FESEM were done to analyze the morphology, structure, and properties of the synthesized composites as well as the chemical interactions of αFe_2_O_3_ with GO. The photocatalytic performance of two synthesized composites was compared with different concentrations of αFe_2_O_3_-GO. The results showed that powder nanocomposites are more effective than thin-film composites for the removal of RhB dye. αFe_2_O_3_-GO-5% powder nanocomposites removed over 64% of dye while thin-film nanocomposites had less removal efficiencies with just under 47% removal rate. The reusability test was done for both materials in which αFe_2_O_3_-GO-5% powder nanocomposites removed a higher rate of dye (up to 63%) in more cycles (6 cycles).

## Introduction

Rapid urbanization, agricultural and industrial modernization increase pollutant concentration in urban sewage sludge and water toxicity, which finally causes infection and disease in different species including human^1–5^. Among all water contamination, colored effluent of the textile industry such as organic dyes pose more environmental threats because they are highly water-soluble and difficult to remove by traditional methods.

Rhodamine B (RhB) is one of the most conventional xanthene dyes, which is commonly used in dyeing and printing of fibers, papers, leathers and alike due to its low cost and high stability^6–8^. It not only has a proven toxic effect on the human body such as causing cancer or birth defect but has also shown a harmful influence on the rate of photosynthesis by preventing light penetration into the water^8^. There are numerous methods for degradation of Rhodamine B and treating of the contaminated water some of which include electrochemical oxidation. However, these approaches only convert organic compounds to carbon dioxide and less harmful molecules, which remain in the water as a second-pollutant. Also, these mentioned methods can consume a huge amount of energy and can be cost-intensive^1^.

The photocatalytic process is one of the effective methods to address the issues due to its effectiveness, low cost, environmentally friendly process, and producing nontoxic components. This system is based on semiconductor materials such as metal oxides (TiO_2_, Fe_2_O_3_, WO_3_, ZnO, Bi_3_O_3_, MnO_2_, SnO_2_) and metal sulfides (ZnS, CdS, MoS_2_, ZnS_2_). By applying an external irradiation light source equal to or higher than a semiconductor band gap, electrons on the valence band of semiconductors would be excited which helps the oxidation and reduction process in different solutions. Degradation of hazardous organic compounds is performed by two radical groups ^•^OH and ^•^O_2_^‾^ which are generated on the surface of semiconductor with the help of produced electron–hole pairs.

α-Fe_2_O_3_ with an indirect bandgap of 2.2 eV has been known as an n-type semiconductor and is applied in a wide range of applications such as catalysis, gas sensors, and solar cells^9–11^. An ideal semiconductor has a considerable bandgap excited with visible light that is environmentally, economically, and practically easy to produce for large-scale field applications. Moreover, hematite can also be effectively used in photoelectrochemical reactors because the valence band edge of α-Fe_2_O_3_ is considerably lower than water oxidation potential^12^. The photocurrent density of hematite is reportedly 12.6 mA/cm^2^ under 1.5 G visible light irradiation^13^. Therefore, hematite is considered a promising material for photoelectrochemical process. However, the higher rate of recombination, lower diffusion length of holes and poor conductivity of hematite cause many problems in photocatalytic processes, which have to be solved^14–16^. In this regard, the photocatalytic activity of hematite has been widely improved in combination with different composite materials such as graphene and graphene oxide (GO)^17,18^.

Graphene, a 2D carbon sheet known as the best candidate in carbonaceous materials, has unique physical, thermal, mechanical, and chemical properties for the fabrication of graphene-based semiconductors^19,20^. In fact, graphene is considered an important material in wastewater treatment technologies because of its outstanding features^3,21,22^. Up to now, graphene and its derivatives are used as a matrix of metal/metal oxide materials to enhance the catalytic performance of materials, including Ni^19^–NiO^23^ for dye-synthesized solar cell, CuO^24^ for catalysts, Pd–Ag^25^ and AuNPs/MoS_2_^26^ for the electrochemical sensor, TiO_2_^27^ and MoS_2_^28^ for electrode, AgBr^29^and Fe_3_O_4_^30^ for water purification, or water splitting^19^. Yuan et al. suggested a new mechanism for the improvement of photocatalytic activities with graphene. They explained that π–π interaction between graphene and benzene rings of the dye molecule is the reason for optimizing photocatalysis in which photo-generated electron can be carried easily from the conduction band to the surface of graphene and the molecules of the decomposed dye. Accordingly, they showed that the photocatalytic performance of graphene mixed with MoS_2_ is two times more than bare MoS_2_^31^. Muthukrishnara et al. used a-Fe_2_O_3_/GO nanorod as efficient photocatalyst to remove dye (methylene dye). They proved that the presence of reduced GO is very effective in dye degradation. The graphene showed the capability of accepting more electrons which reduced recombination rate and therefore increased dye removal rate^32^. Moreover, Zhang et al. designed α-Fe_2_O_3_/GO powders have been used to degrade rhodamine 6G, methyl orange, methylene blue and bisphenol amine. Due to the significant increase in surface area, the introduction of GO enhanced the degradation of mentioned dyes. Besides, their results showed that the synthesized nanocomposite had more stability with a removal efficiency of up to 90% after ten cycles^33^.

The morphological structure of photocatalysts has shown an effective role in the improvement of photocatalytic efficiency^34^. In the slurry system, photocatalytic powder nanocomposites are more prone to react with toxic materials due to having more contact with the pollutants. This is considered one of the easy reactor operation methods, which is significantly active for water detoxification. However, the complexity of nanoparticles recovery after reaction and low efficiency makes this process more difficult to use in large scales. Hence, recent studies have focused on finding alternative morphologies to tackle these problems. In this regard, immobilization of hematite on a conductive support layer by using a potential bias has been introduced as a photoelectrocatalysis^35^.

Herein, a comparison is made between GO/ α- Fe_2_O_3_ as a photoelectrocatalytic thin film and GO/ α-Fe_2_O_3_ as a powder nanocomposite to investigate the best morphological structure for degradation of rhodamine B in textile wastewater. In this regard, the morphology and structure of both synthesized nanocomposites and thin films were fully investigated with different analytical devices. The photocatalytic performance of both materials was tested under the same condition for the first time. Dye concentration and pH were fixed at its optimum value (natural condition) and nanocomposites concentration was varied in the range of 2–8% to study its contribution on the rhodamine B photocatalytic degradation efficiency. The reusability of synthesized materials was also tested to evaluate the capacity of these nanoparticles with different structure as a thin film or powder nanocomposite.

## Experimental procedure

### Materials

Graphite powder, Sodium nitrate (NaNO_3_), Sulfuric acid (H_2_SO_4_), potassium permanganate (KMnO_4_), hydrochloric acid (HCl), lithium perchlorate (LiClO_4_), hydrogen peroxide (H_2_O_2_), iron II chloride tetrahydrate (FeCl_2_. 4H_2_O), iron III chloride hexahydrate (FeCl_3_. 6H_2_O), Fluorine-doped Tin Oxide (FTO) (SnO_2_: F), and ethanol (C_3_H_8_O) were obtained from Merck Co. (Darmstadt, Germany). Ethanol and deionized water were used during all experiments as a solvent for performing experiments and washing the products. All chemicals were of the analytical grade and used directly without further purification.

### Preparation of GO/α- Fe_2_O_3_ layers

#### Thin film reduced GO

In order to synthesize a thin layer of reduced GO onto the FTO substrate by electrodeposition, the 2.5 mg/ml solution of GO synthesized by a modified Hummers method^21^ was provided. In this regard, the three electrode cells were used with Ag/AgCl as a reference electrode and FTO as a counter electrode at a rate of − 20 mV/s, which is optimum potential to obtain uniform and transparent films. During this operation, reduction and deposition of GO sheets onto the FTO substrate were attained by oxygen-containing functional groups on the GO.

#### Hematite deposition onto the GO film

**Figure 1:**
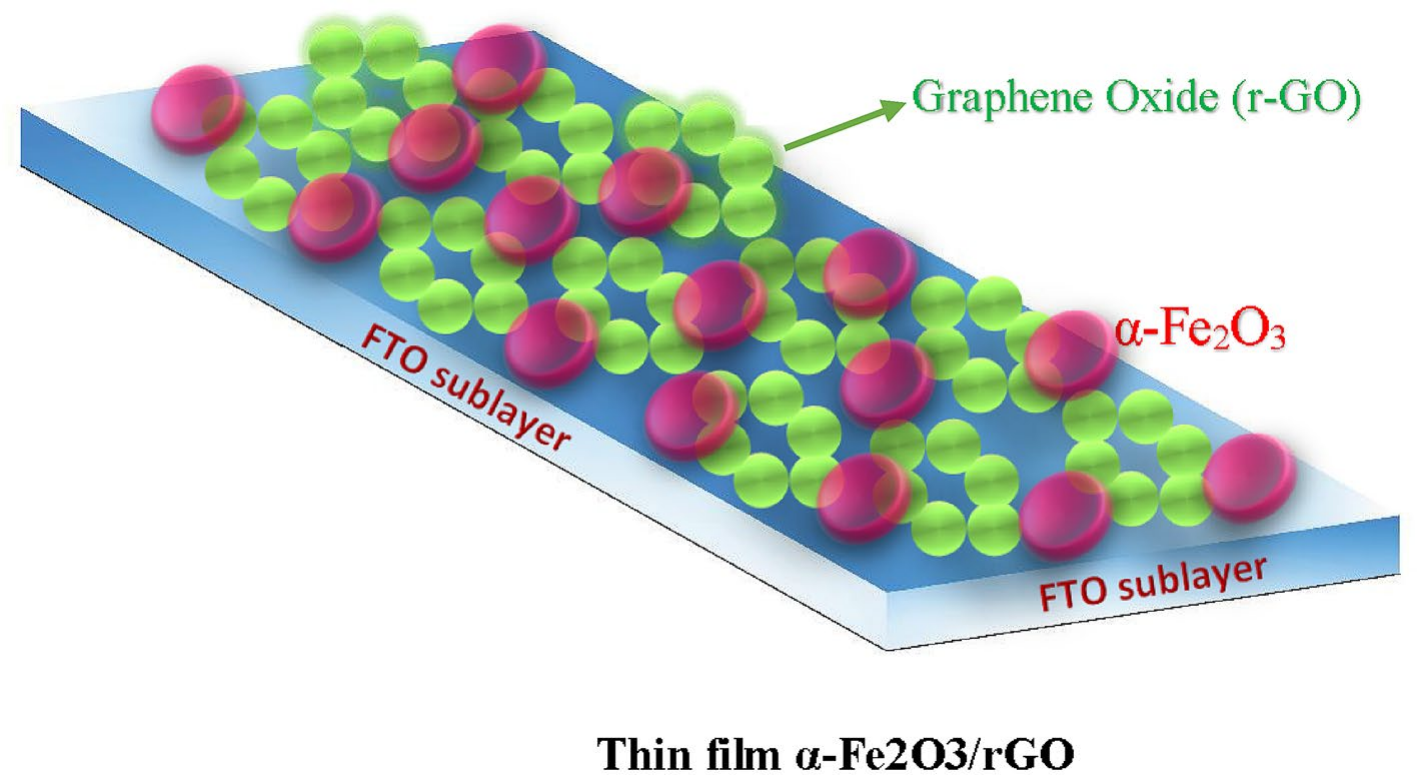
Synthesis of thin-film α-Fe2O3/rGO on the FTO substrate.

The electrodeposition was performed using an aqueous solution containing 0.02 M FeCl_2_.4H_2_O. The pH of the solution was adjusted to 3.4 at 70 °C with constant potential. Before the deposition, the GO film was annealed at 200 °C for 20 min, due to increased adhesion of GO to the substrate (FTO). After that, a new substrate (G-FTO) was used as a working electrode, while FTO was used as the counter electrode. The reference electrode was an Ag/AgCl electrode. The optimum deposition potential of 1.2 V was found to supply uniform films with the highest transparency. After deposition, the resulted film was rinsed with deionized water and submitted to the atmospheric thermal treatment at 500 °C for 30 min. Figure 1 shows the schematic illustration of the synthesized thin-film α-Fe_2_O_3/_GO on the FTO substrate.

### preparation of nanocomposite GO/ α-Fe_2_O_3_

1.35 g FeCl_3_.6H_2_O was dissolved in 50 ml distilled water and stirred vigorously for 20 min. Subsequently, 0.05 g GO, which was synthesized from natural graphite powder by modified Hummers method, was dispersed into the 50 ml ethanol by ultrasonication for 30 min to get a homogenous suspension, and then added into the iron III chloride hexahydrate solution. Via thermostatic water bath, the aqueous solution evaporated and the paste sediment remained. The precipitate was rinsed with distilled water and ethanol three times and collected by centrifugation after each wash cycle at 4000 rpm. The resulting black solid products were dried at 70 °C in a vacuum oven. When the products were cooled to room temperature, they were calcined at 500 °C with a ramping rate of 3.5 °C /min and kept at 500 °C for 1 h. For comparison, Fe_2_O_3_ nanoparticles were synthesized by a similar procedure with no GO added. Moreover, different weight percentages of GO (2, 5, and 8%) were prepared which are referred as 1-GO-α-Fe_2_O_3_, 2-GO-α-Fe_2_O_3_, and 3-GO-α-Fe_2_O_3_, respectively. It should be noted that higher amount of GO can increase the rate of electron-holes recombination by enhancing the chance of collision. Besides, more GO content may act as a shield for the xenon-lights which is absorbed by the active sites of catalyst. This effect is called a “shielding effect” which consequently deteriorates photocatalytic activity. Thus, the proper loading amount of GO is crucial for improving photocatalytic properties, which is 2–5 wt % based on previous works.

### Characterization

X-ray diffraction (XRD) analysis for phase identification was carried out by Philips X-Ray Diffractometer PW 3710 with CuK_α_ radiation (ƛ = 1.54 Å) at 50 kV and 250 mA in the range of 2θ value from 10° to 80°. Fourier transform infrared (FTIR) spectra (PerkinElmer, Spectrum 400) was recorded with a diffuse reflectance attachment over a range of 400–4000 cm^−1^ to detect the present functional groups. The morphological properties were also examined via Field Emission scanning electron microscope (FESEM, Cambridge 360) coupled with energy dispersive X-ray spectroscopy (EDX). Calculation of the adsorbed amount and specific surface area, and measurement of sorption isotherm and pore size were obtained by BET analysis (BELSORP-mini II). Atomic force microscopy (AFM, Auto Probe CT) with a silicon needle of 10 nm tip radius in contact mode in air was performed to calculate GO sheets thickness. The sample (powder nanocomposite) was dispersed in doubled distilled water and a drop of suspension was placed on newly cleaved mica surfaces and dried in air. Raman scattering spectra were acquired by Raman spectrometer (SENTRRA, Germany). The surface chemical composition of GO/α-Fe_2_O_3_ films was analyzed by X-ray photoelectron spectroscopy (XPS, Al-Kα, 1486.6 eV). The accuracy of the measured data was checked by calibration of binding energy with the sp^2^ C 1 s band at 285.0 eV. The binding energy was evaluated by the hemispherical energy analyzer (Specs EA 10 plus) in a vacuum. The SDP software (version 4.1) with Gaussian—20% Lorentzian peak fitting was used for peak separation, background subtraction, and curve fitting. The UV–vis spectra were determined for both dilute dispersions of nanocomposite powder sample and film in colored and bleached stated by UV–vis spectrophotometer (Perkin Elmer) in the wavelength range between 350 and 800 nm.

### Photocatalytic property

#### Powder nanocomposite

The efficiency of the synthesized photocatalyst was evaluated by the removal efficiency of RhB under visible irradiation (75-W xenon lamp with cutoff UV light filter). 0.02 g of various weight percentages of GO/α-Fe_2_O_3_ nanocomposites were suspended in 10 ml distilled water. 20 ml of each suspension was then added to the 20 ml of RhB (25 mg/L) aqueous solution in neutral pH. Before irradiation, suspensions were stirred vigorously in a dark and isolated room for 60 min to measure the rate of adsorption of each sample. Mixtures of dye- GO/α-Fe_2_O_3_ powders nanocomposites were exposed under visible irradiation in different step times (45, 75, 90, 120 min) following which about 5 ml samples were collected and immediately analyzed by UV–vis spectrophotometer. All steps were performed on the same day.

#### Thin film nanocomposite

**Figure 2:**
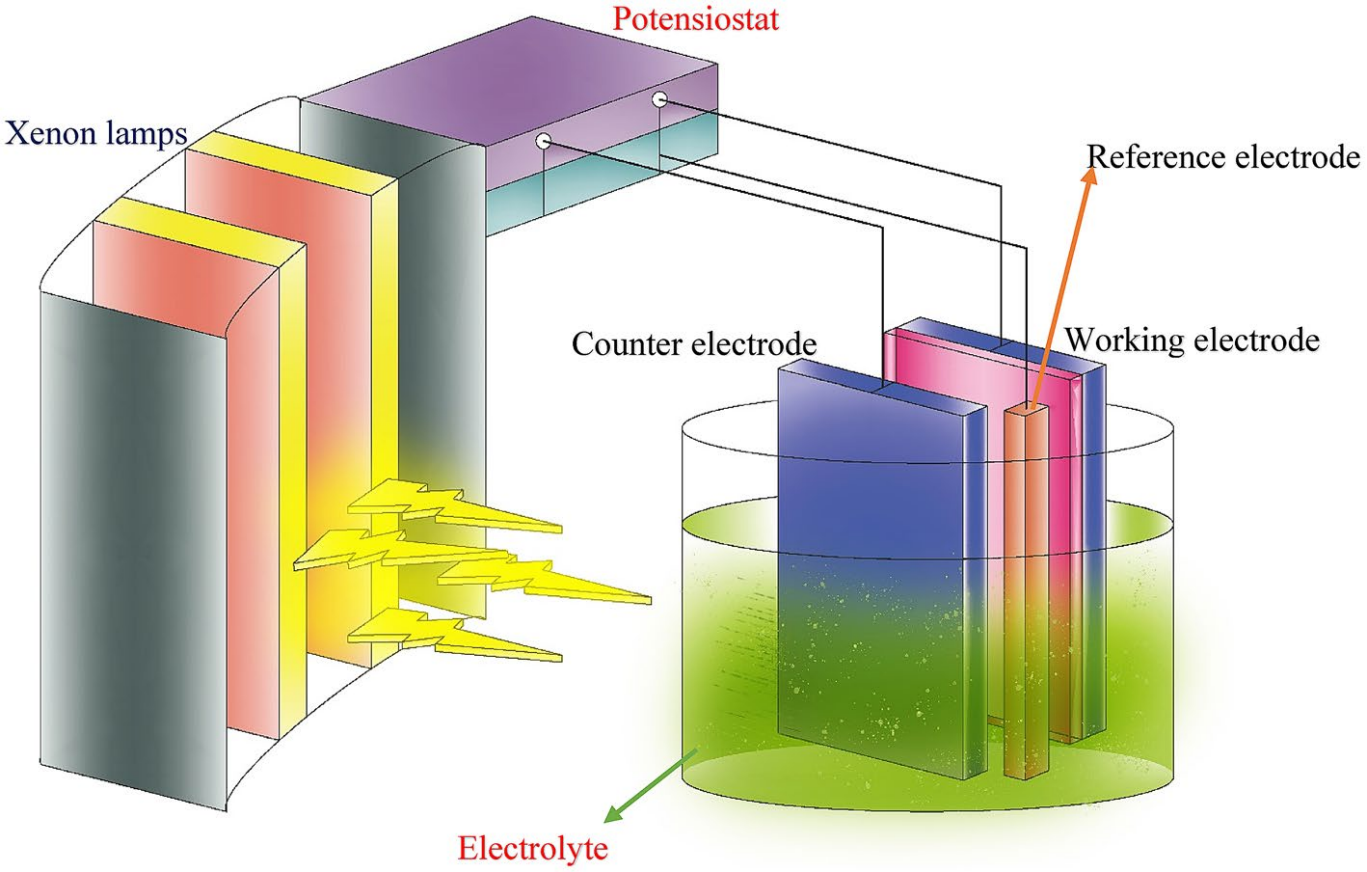
Schematic of photoelectrochemical reactor.

The photoelectrocatalytic degradation process for thin film was done in a reactor containing working electrode, counter electrode, and electrolyte. The GO/α-Fe_2_O_3_ thin film deposited on the FTO glass was the working electrode, FTO glass was counter electrode and Ag/AgCl was the reference electrode in the reactor. The photoanode electrode was illuminated by the visible light source. The potentiostat was used to apply 1.5 V as an external voltage for the increase in the photocatalytic reaction rate. Dye solution (25 mg/L) was injected into electrolyte and photoelectrocatalytic test was done at the interval of 0, 30, 60, 90 and 120 min. After each testing process, dye solution was circulated again using peristaltic pump to measure the reusability of thin layers. Figure 2 illustrates the schematic diagram of photoelectrocatalytic process in the presence of an external visible light source. When xenon light hits the surface of thin films, the excited electrons and holes form the radicals such as O_2_° or OH°. These radicals can degrade dyes near the surface of working electrode and turn them into harmless materials. The created voltage helps the reaction rate in the photocatalytic process and therefore, enhances the dye removal efficiency^36,37^.

## Results and discussion

### XRD analysis of nanocomposite

**Figure 3:**
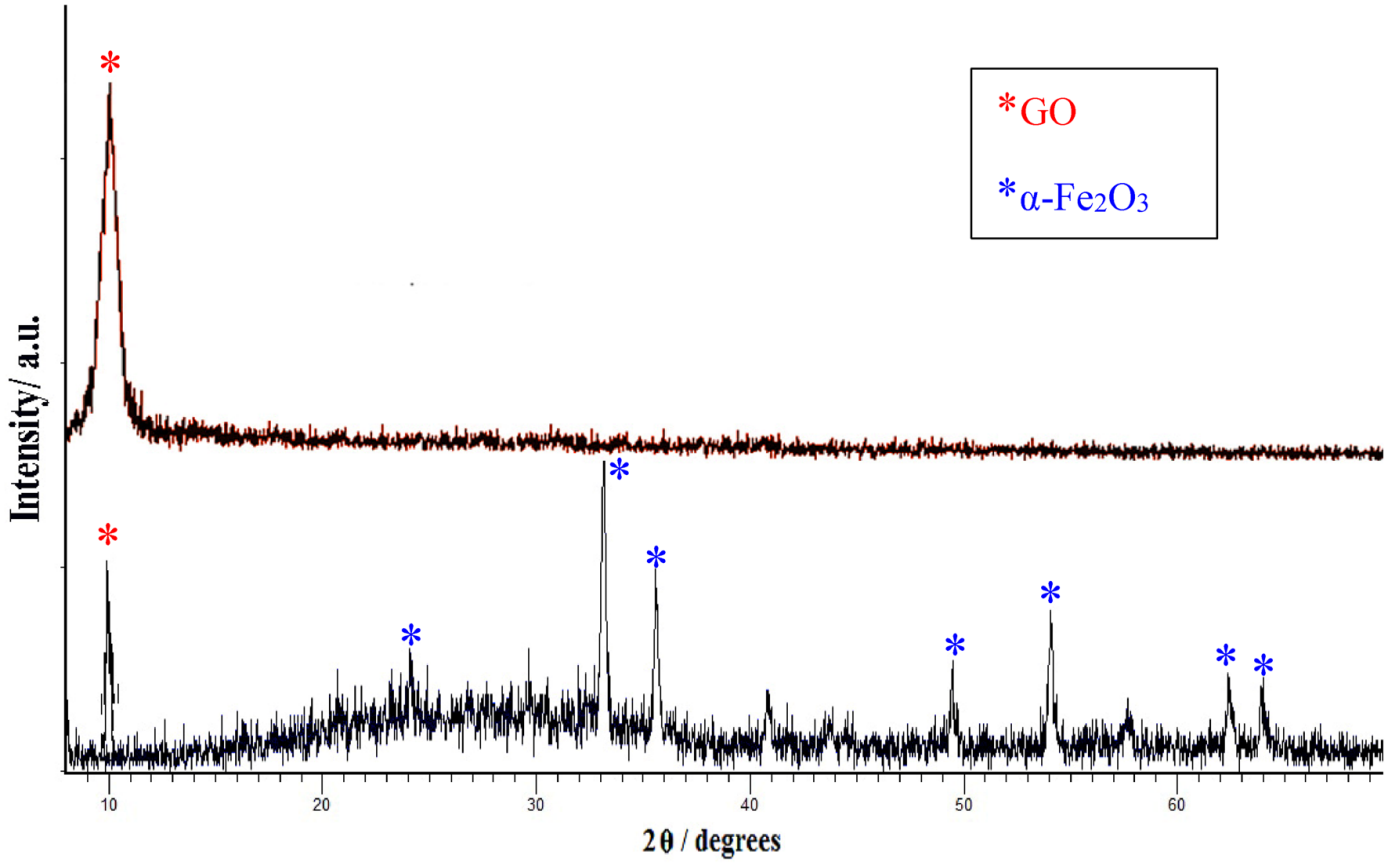
XRD analysis of GO and GO-Fe_2_O_3_ powder nanocomposites.

Figure 3 shows the XRD spectrum for the as-prepared GO and nanocomposite powders GO/α-Fe_2_O_3_. The peaks were shown with symbol (●) located at 23.25°, 33.18°, 35.75°, 40.89°, 49.52°, 54.14°, 57.52°, 62.49°, 64.07° correspond to standard data of pure α-Fe_2_O_3_ diffraction peaks (reference code 01-079-1741). These peaks ascribe to the rhombohedral crystal structure of Hematite with a lattice parameter of a = b = 5.03 Å and c = 13.74 Å. The XRD pattern of GO and GO/α-Fe_2_O_3_ shown in Fig. 3. The XRD of GO with symbol (*) displays a characteristic peak at 10.37° with an index of (001) defining 0.83 nm d-spacing which is increased from 0.34 nm interlayer spacing of graphite. This specific peaks also could be seen in GO/ α-Fe_2_O_3_ XRD analysis (with * mark) which indicates no transformation occurred on GO sheets during synthesis as well as that confirms existence of graphene oxide in GO-α-Fe_2_O_3_ nanocomposite. This significant increase in d-spacing of GO is assigned to the presence of oxygen functional groups such as carboxyl groups (COOH–), hydroxyl groups (OH–), epoxy groups (C–O–C) that can be confirmed by FTIR. The identification of GO peaks was hardly recognized from the composite pattern which might be explained by the minor amount of GO (α-Fe_2_O_3_ − 0.05% GO) in nanocomposite and high dispersion of GO-sheets among Fe_2_O_3_ nanoparticles.

According to the Debye-Scherrer equation, the particle size of hematite was obtained from the XRD pattern; $D=\frac{{K \lambda}}{{\beta cos\theta }}$ (where K = 09, ƛ = 0.154 nm, β = the reflection width (2θ) and θ = the Bragg angle). The result shows that the average crystal size is about 48.27 nm.

### FTIR spectrum of nanocomposite

**Figure 4:**
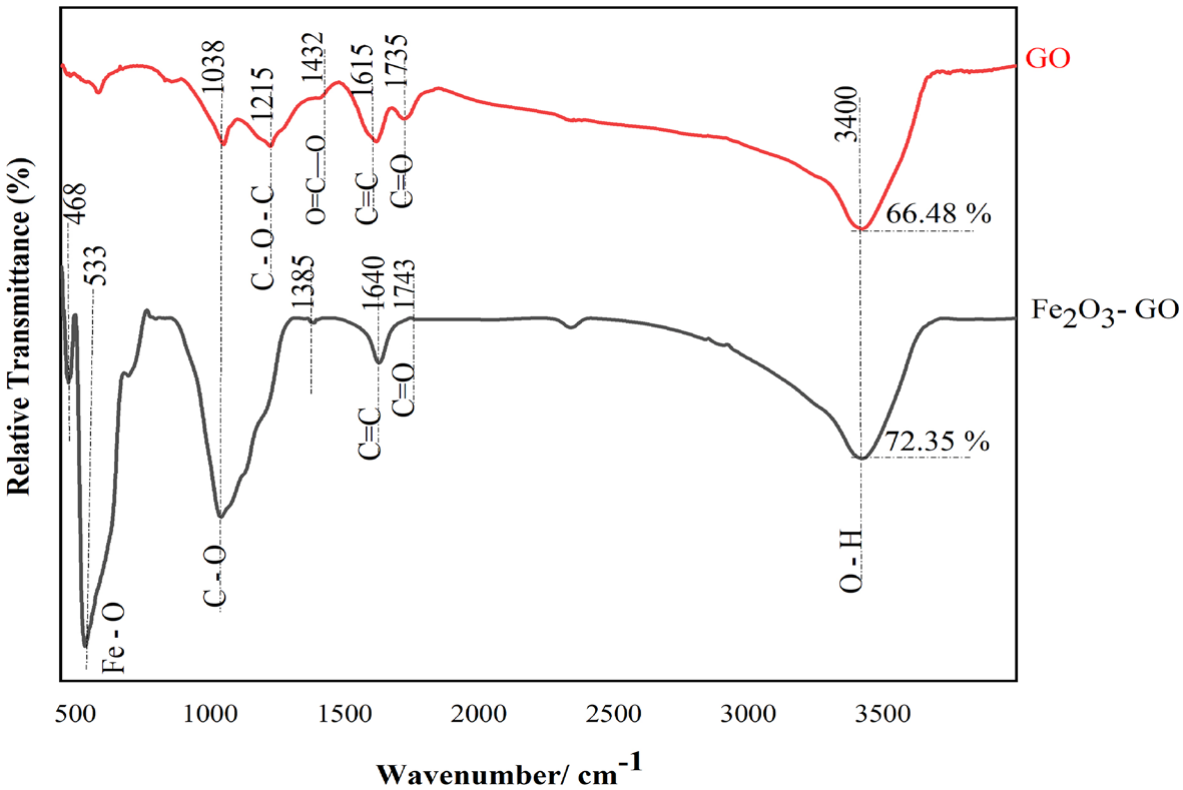
FTIR analysis of GO and Fe2O3-GO powders.

FTIR spectra of the nanocomposites (Fig. 4) were obtained under transmission mode to identify the functional groups of compounds presented in GO-α-Fe_2_O_3_ composite. Spectrum of GO confirmed the presence of the alkoxy C–O stretching vibrations (1038 cm^−1^), epoxy C–O–C stretching modes (1215 cm^−1^), carboxyl O=C–O (1432 cm^−1^), and aromatic C=C skeleton vibrations (1615 cm^−1^). The C=O stretching band, located at the edge of GO sheets, was emerged at 1735 cm^−1^. All these functional groups could be seen in GO-Fe_2_O_3_ spectrum, however the position of bonds are red shifted and sharpness of the peaks is changed indicating the change in the coordination environment of various functional groups in GO-α-Fe_2_O_3_. The band located at 3400 cm^−1^ is assigned to the O–H stretching vibration of C–OH groups, in which this broad absorption is decreased in GO-α-Fe_2_O_3_ spectrum due to the reduction of GO during heat treatment and restoration of the conjugated aromatic system. As well as that after coating with α-Fe_2_O_3_, the C=O stretching band at 1743 cm^−1^ become weaker than GO because of the formation of –COO–. Compared with GO, the absorption bands at 468 cm^−1^ and 533 cm^−1^ are ascribed to Fe–O stretching mode, which verify the existence of α-Fe_2_O_3_ chemical compounds attached to the –COO on the edge of GO nano sheets. The next clue for the formation of monodentate and bidentate ligands in a complex between Fe and the carboxyl group is the additional vibrational band at 1385 cm^−1^, confirming the covalent bond formation between hematite and GO^38^.

### Raman spectra of nanocomposite

**Figure 5:**
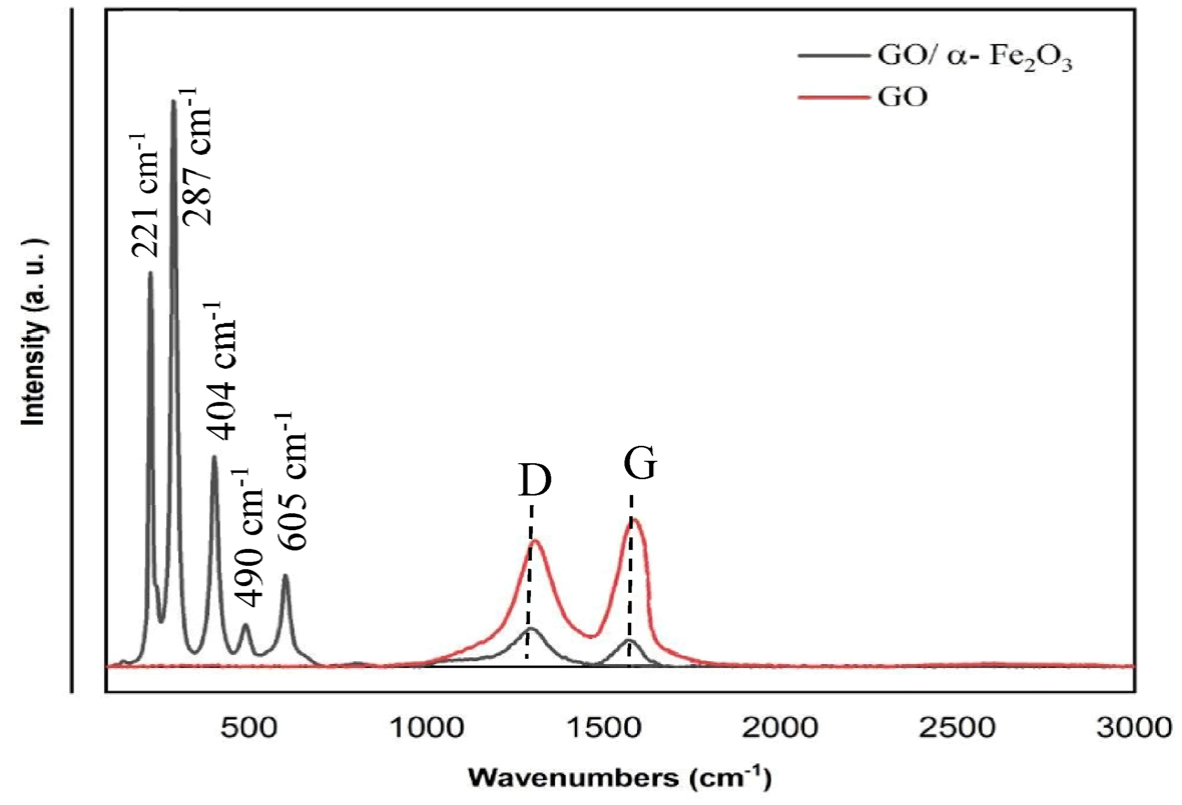
Raman spectra of GO and GO/α-Fe2O3.

Characterization for structural properties of carbon material including defect density and disorder structures is investigated by Raman spectroscopy (Fig. 5). The Raman spectroscopy of GO revealed two prominent peaks at 1304 and 1582. The D-peak that corresponds to the breathing modes of carbon sp^2^ rings (κ-point phonons of A_1g_ symmetry) required defects and disordered atomic arrangement for its activation. The G-peak corresponds to the E_2g_ phonon of carbon sp^2^ atoms. These two peaks of GO also could be recognized in Raman spectroscopy of GO-α-Fe_2_O_3_ as well as fundamental Raman vibration of α-Fe_2_O_3,_ suggesting that the structure of GO remained in the composite. In an additional analysis of the Raman spectrum, the presence of strong peaks at 221, and 287 cm^−1^ (A_1g_ symmetry), and weaker peaks at 404, 490, and 605 cm^−1^ (E_g_ symmetry) show the formation of hematite phase accurately. It can also be observed that the I_D_/I_G_ ration elevated from 0.865 for GO to 1.412, which confirms penetration of Fe_2_O_3_ nanoparticles between GO layers as well as increasing disorders and defects in GO sheets^39^.

### Microscopy analysis of nanocomposite

**Figure 6:**
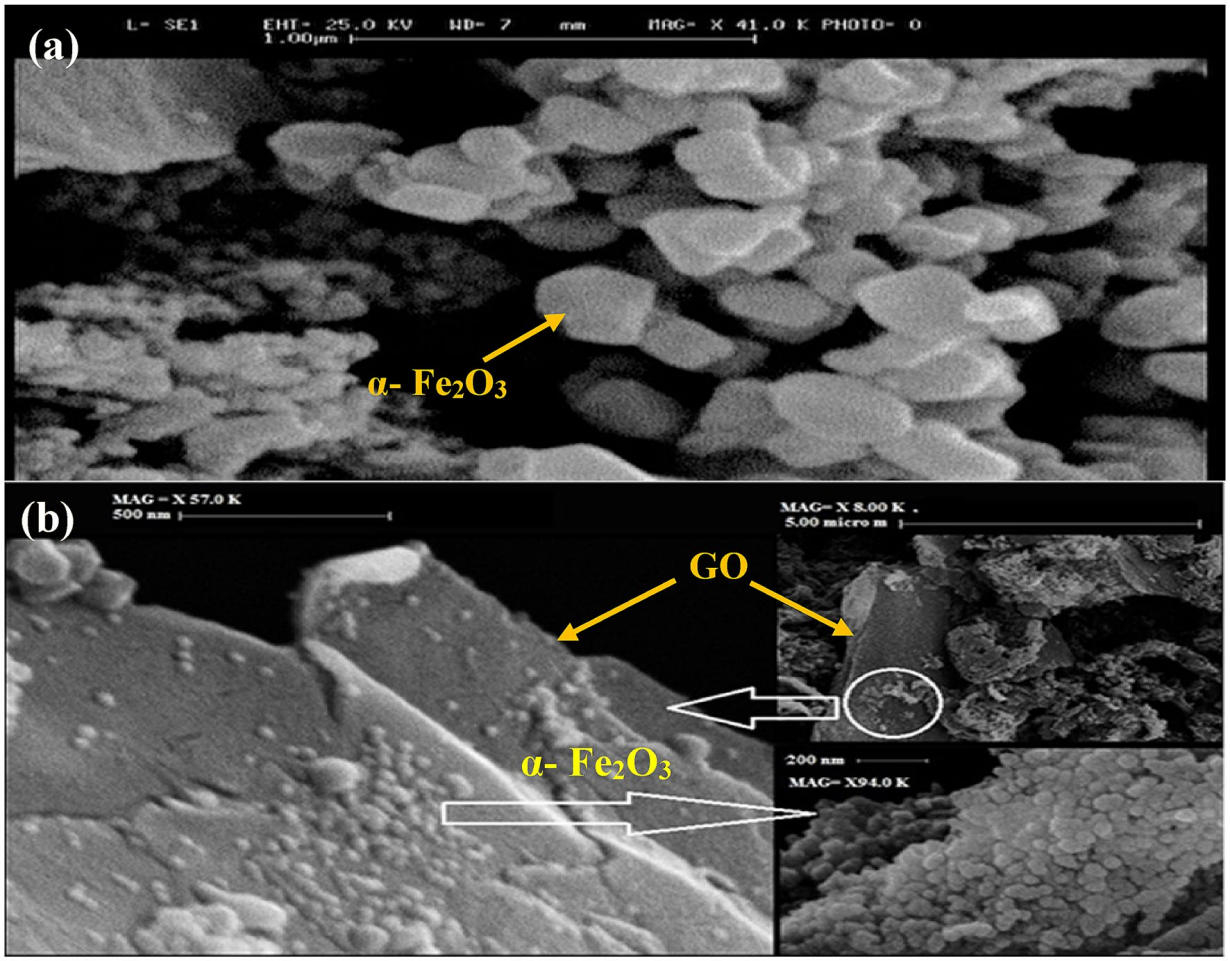
FESEM analysis of nanoparticles of α-Fe2O3 (**a**) and nanocomposite α-Fe2O3-5% GO (**b**).

The detection of morphology, size of particles, shape, and thickness of nanocomposite were done by FESEM and AFM analysis. The FESEM images of nanoparticles of α-Fe_2_O_3_ and nanocomposite α-Fe_2_O_3_-5% GO are shown in Fig. 6. These images provide clear evidence that α-Fe_2_O_3_ particles with the average size of 63 nm were uniformly distributed on GO nano sheets. These well-dispersed hematite nanoparticles provide direct interaction with GO surface, which is desirable for photocatalytic application. The thickness of the exfoliated GO sheets was measured by AFM, which have a sharp edge with a thickness of 2.5 nm. The typical thickness of monolayer GO sheets is in the range 0.7 nm. Thus, it might be a four-layered GO sheet.

#### Characterization of α-Fe_2_O_3_/GO thin film nanocomposites

**Figure 7:**
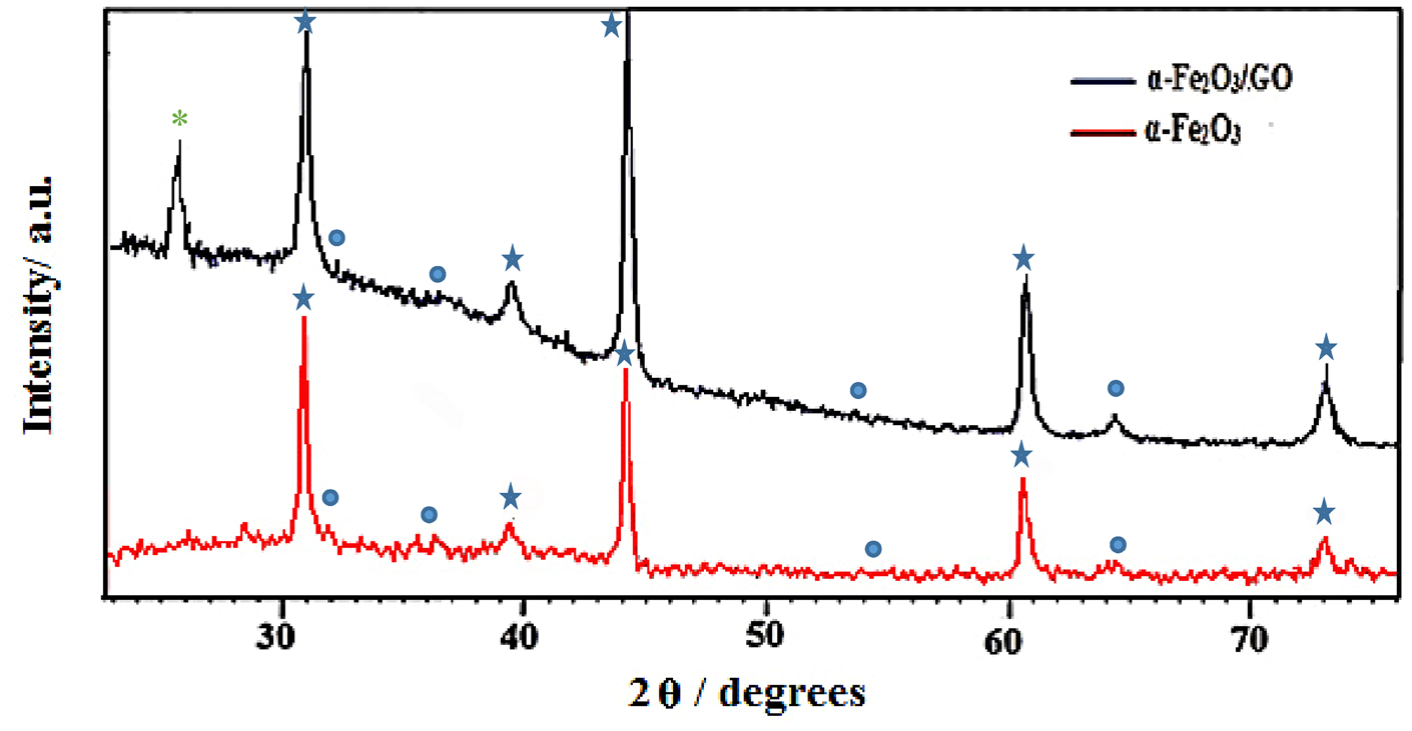
XRD analysis of synthesized thin film layer.

X-ray diffraction patterns was done for identifying the composite structures on the FTO substrate. As it is clear in Fig. 7, the identified peaks at 32.3.3°, 35.9°, 54.1° and 63.6° shown by symbol (●) are contributed to the crystallographic directions of the α-Fe_2_O_3_ phase. Diffraction peaks at 31.1°, 39.2°, 44°, 60.9° and 73.1° with symbol (*) show the presence of FTO substrate in the nanocomposite. The XRD also confirmed the existence of GO (*) on the FTO substrate at peak 10°.

**Figure 8:**
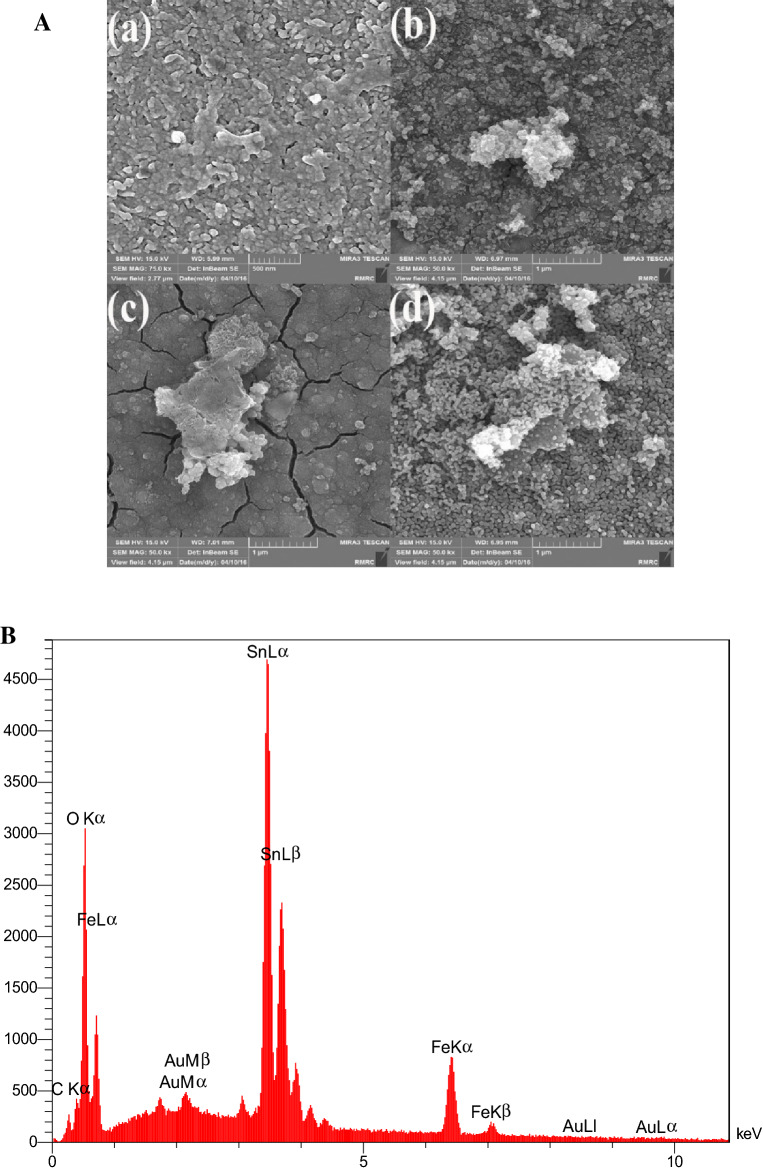
(**A**) FESEM analysis (**a**) Fe2O3-GO annealed at 300 °C (**b**) Fe2O3-GO annealed at 400 °C (**c**) Fe2O3-GO annealed at 500 °C (**d**) Fe2O3-GO annealed at 700 °C and (**B**) FESEM-EDS mapping (annealing at 500 °C) of synthesized thin film layer.

Figure 8 shows the morphology of GO/α-Fe_2_O_3_ thin films which was annealed at different temperatures of 300, 400, 500, and 700 °C. Based on the FESEM analysis of GO/ α-Fe_2_O_3_ thin films after annealing at 300, 400, 500 and 700 °C, the uniform distribution of iron oxide nanoparticles on the surface of graphene layers was occurred. The corrugated and crumpled morphology of GO nanosheets is clearly seen in all synthesized GO/α-Fe_2_O_3_ thin films. Increasing the temperature resulted in the formation of a higher rode shape structure in the layers. The perfect synthesis of α-Fe_2_O_3_ nanoparticles with GO nanosheets occurs in most part of layers, which proves the reduction of agglomeration in GO nanosheets and decrease in the aggregation of nanoparticles. The more combinations of nanoparticles and GO nanosheets on the FTO substrate may lead to higher specific surface area. According to the FESEM analysis in higher temperatures (Fig. 8Ac,Ad), it seems that the nanoparticles have diffused into the sub layer resulting in the size of nanoparticles to seem smaller on the surface. The FESEM-EDS mapping of all presented elements in the thin-film layer is shown in Fig. 8b. The result of the analysis shows that Fe and O elements were equally diffused on the surface of GO nanosheets.

#### Quantitative result

**Figure 9:**
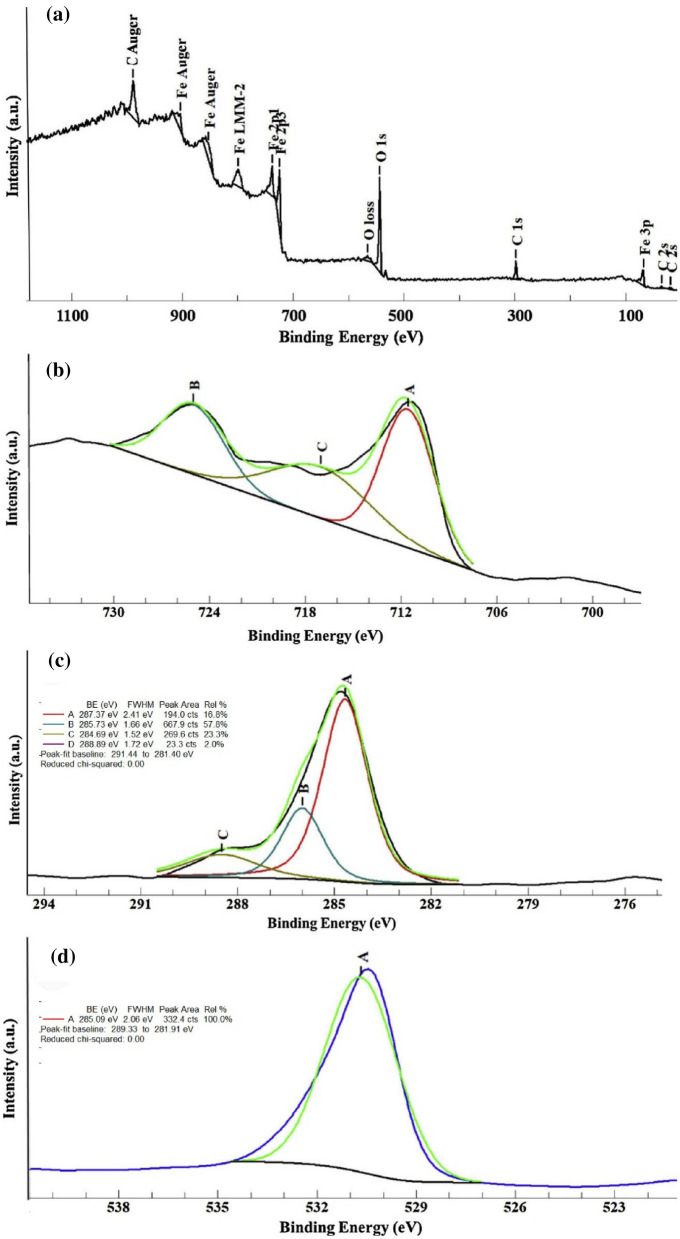
XPS analysis of synthesized thin film layers for identification of Fe (**b**), C(**c**) and O (**d**) orbitals.

XPS analysis was done to identify the surface chemical composition of thin-film Fe_2_O_3_/GO^40^. The Fe orbitals (2p), O (1 s), and C (1 s) were shown in the XPS spectrum of synthesized thin-film nanocomposite at 700 °C. According to Fig. 9, the XPS peaks Fe 2p_3/2_ and Fe 2p_1/2_ are located in the binding energy of 711.1 and 725.1 eV, respectively. The satellite peak of Fe 2p_3/2_ is approximately 8 eV higher than the main peak. Here, the satellite peak is close to 718.7 eV. The Fe 2p_3/2_ peak is narrower and stronger than Fe 2p_1/2_ peak. Moreover, the area of Fe 2p_3/2_ is greater because of spin–orbit (j–j) coupling. XPS analysis also showed three peaks for C (1 s) including non-oxygenated aromatic carbons with orbital sp2 at 284.67 eV, oxygenated functional group C–O at 286.00, and C==O at 288.5 eV. The existence of anionic oxygen in Fe_2_O_3_ and functional oxygen groups in GO sheets created the binding energy of 531.26 eV which is for O (1 s) peak (Fig. 9d).

### Photocatalytic test of α-Fe_2_O_3_/GO nanocomposites and α-Fe_2_O_3_/GO thin films

**Figure 10:**
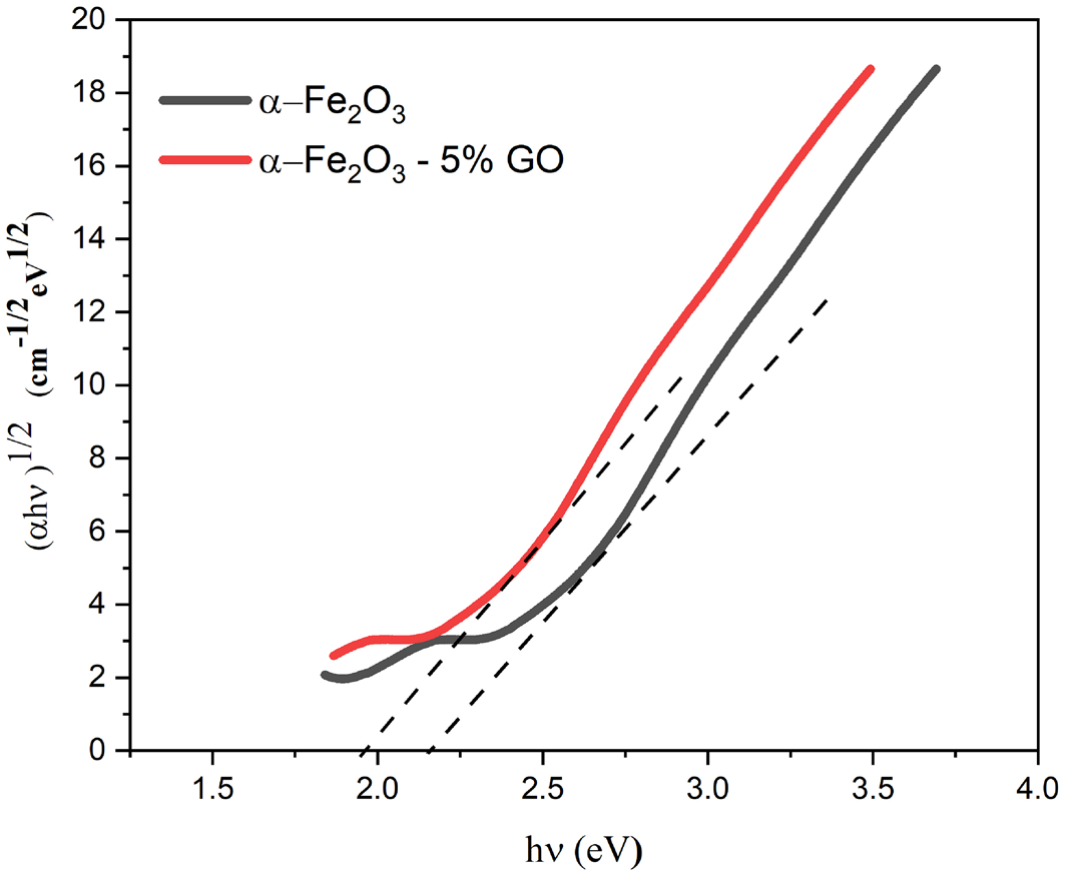
Tauc plot of α-Fe_2_O_3_ and α-Fe_2_O_3_- 5% GO.

**Figure 11:**
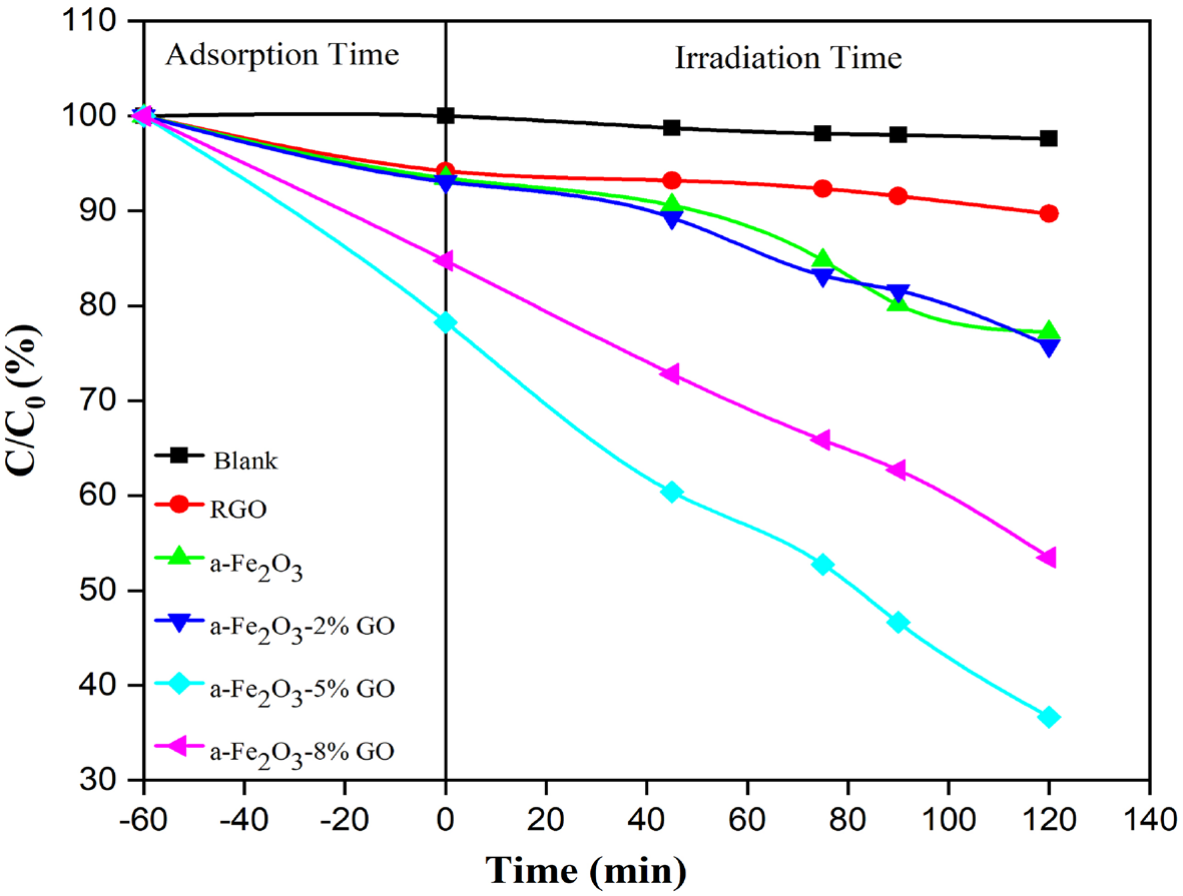
The photocatalytic result of all powder nanocomposites with pure Fe_2_O_3_.

The photocatalytic properties of the nanocomposites and thin film layers were tested using irradiation of xenon lamp (185–2000 nm). The nanocomposites were initially tested under different pH values varied from 2.0 to 10. The dye removal was high at a wide range of pH. The reason being that when α-Fe_2_O_3_/GO is applied in decolorization process, the catalytic process of dye solution containing water molecules with the increased lattice metal ion sites on the surface of α-Fe_2_O_3_ creates surface hydroxyl groups. Even at a low pH (pH = 3–5), the α-Fe_2_O_3_ nanoparticle surface still has a low -OH content. Thus, the dissolution behavior is inhibited and the catalyst remains active over a wide range of pH with high degradation efficiency^41,42^. Hence, in this study, natural condition (pH = 7) was considered for all further photocatalytic testing processes. As for α-Fe_2_O_3_/GO powder nanocomposites, the test was done without irradiation for the first 60 min to evaluate the absorption of nanocomposite in dark. Then, photodegradation was tested for 120 min irradiation. Maximum adsorption and absorption of samples are shown in Tables 1 and 2, respectively. Different concentrations of synthesized nanocomposite were tested which showed excellent dye removal for all of them. Nanocomposites can excite more electrons to degrade dye solution due to its higher surface area, although the recombination rate in α-Fe_2_O_3_ is relatively high and major photocatalytic activity of GO is dedicated to surface adsorption. In this regard, a strong connection between GO and α-Fe_2_O_3_ can facilitate the degradation process and reduce the recombination of electron hole pairs due to high surface area of GO providing copious photocatalytic sites. As well as that because of the strong reaction between negatively charged structure of GO and cationic dyes such as RhB, the photodegradation process increases significantly. In fact, the structure of synthesized nanocomposite delays the combination of electron–hole pairs in the system and therefore increases dye removal efficiency. Higher surface area is another reason for turning dye molecules into harmless products. More photo generated electron–hole pairs participate in degradation reactions, which finally leads to higher dye removal efficiency^43,44^. The gap energy between conduction and valence band in pure α-Fe_2_O_3_ and α-Fe_2_O_3_-GO are respectively calculated to be 2.33 eVand 1.95 eV from tauc illustrated in Fig. 10, which provided by interception with x axis. The reduction in the value of band gap of α-Fe_2_O_3_-GO composite is in good agreement with the hypothesis discussed above. Furthermore, as it is clear in Fig. 11, the pure αFe_2_O_3_ (23% dye removal) and free GO (10% dye removal) are by far lower than all powder nanocomposites especially 5% GO/α-Fe_2_O_3_ with 64% dye removal. The electrons were transferred through sp2 hybrid carbon channels on the GO sheets, which reduced the recombination of electron-holes. Also, the presence of GO increases the light-receiving capacity which leads to the improvement of photocatalytic process.

**Table 1:** Maximum adsorption of different weight percentages of GO nanocomposites during 60 min maintained in dark room.

Sample	GO	α-Fe_2_O_3_	α-Fe_2_O_3_-2% GO	α-Fe_2_O_3_-5% GO	α-Fe_2_O_3_-8% GO
Maximum adsorption	0.0188 g	0.0186 g	0.0184	0.0156 g	0.01694 g

**Table 2:** Maximum absorption of different weight percentages of GO nanocomposites in different step times of exposure.

Sample	Maximum absorption			
	45 min	75 min	90 min	120 min
GO	0.01864 g	0.01846 g	0.01831 g	0.0179 g
α-Fe_2_O_3_	0.01811 g	0.01695 g	0.01602 g	0.01544 g
α-Fe_2_O_3_-2% GO	0.01785 g	0.01664 g	0.01631 g	0.01515 g
α-Fe_2_O_3_-5% GO	0.01207 g	0.01055 g	0.00932 g	0.00733 g
α-Fe_2_O_3_-8% GO	0.01455 g	0.01317 g	0.01253 g	0.01069 g

Photocatalytic activity of α-Fe_2_O_3_@ GO as powder nano composites is compared with similar reports that is tabulated in Table 3. As the results show, the photocatalytic activity of powder produced in this paper is approximately 2 times higher than others, in spite of lower mass of catalyst.

**Table 3:** comparison of photocatalytic performance of α-Fe_2_O_3_@ 5% GO powder nano composite with other reported nanomaterials.

Catalysts	Pollutants	Mass of catalysts (mg)	Concentration (mg/L)	Irradiation time (min)	Degradation efficiency	References
α-Fe_2_O_3_@ GO	MB	100	40	80	40%	^43^
rGSs/Fe_2_O_3_/PPy	MB	100	50	80	33%	^45^
TiO_2_& Fe_2_O_3_@graphene	RB	200	5	140	36%	^46^
N-doped graphene- α-Fe_2_O_3_	Phenol	15	5	90	27%	^47^
α-Fe_2_O_3_@ 5% GO	RB	20	25	120	64%	This work

**Figure 12:**
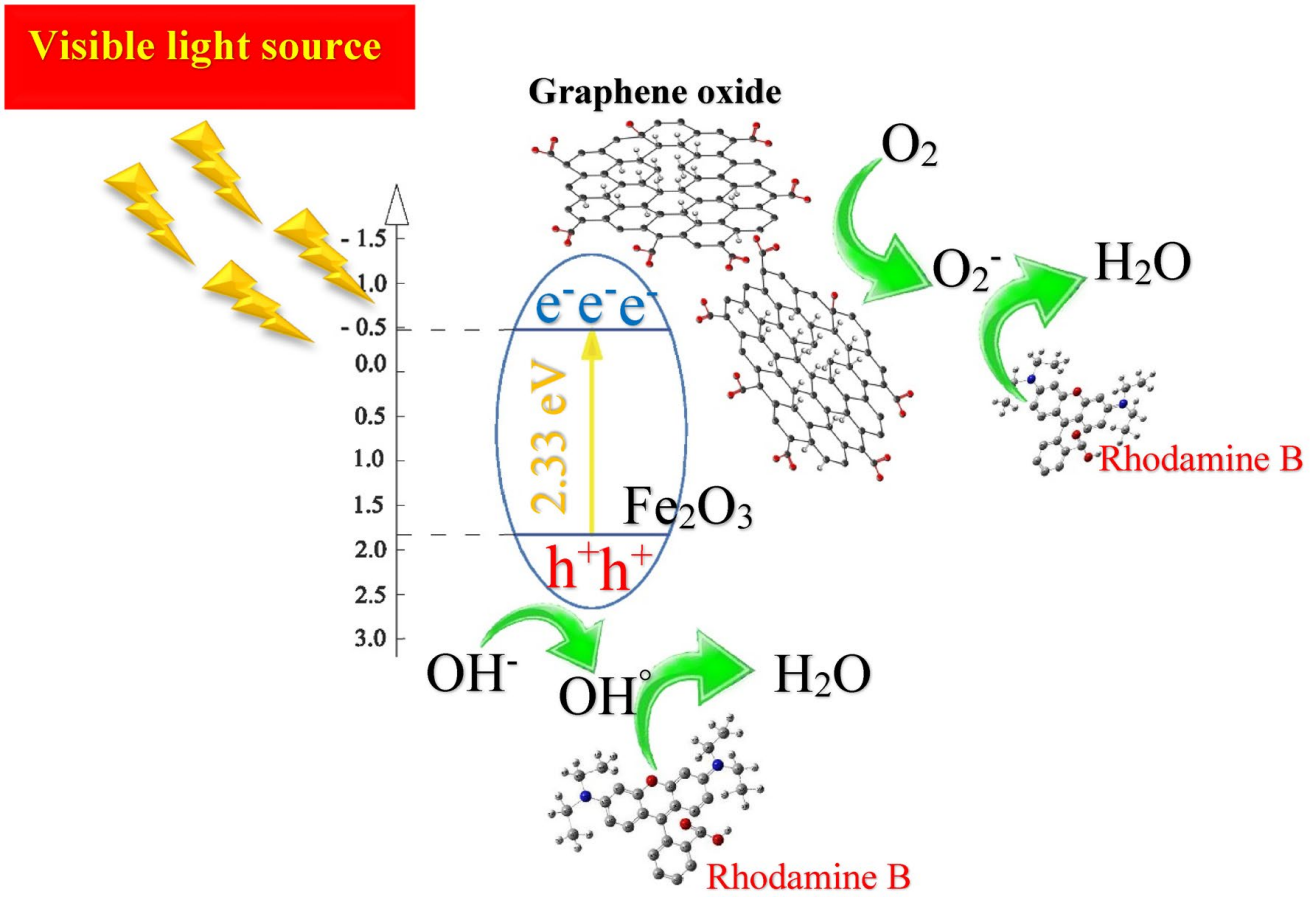
The photodegradation mechanism of RhB with α-Fe2O3/GO thin films.

α-Fe_2_O_3_/GO thin films, on the other hand, showed a lower removal efficiency compared to nanocomposites. The best results were for α-Fe_2_O_3_/GO 5% with just over 47% dye removal. The main reason for this is that the content of nanocomposites involved with dye molecules is limited and therefore lower percentage of dyes is eliminated by the photocatalytic process. Pure Fe_2_O_3_ and free GO showed minimum efficiency compared to other thin film composites. Fe_2_O_3_-GO 8% also indicated acceptable performance with 39.1% removal efficiency after 120 min irradiation. The results are shown in Fig. 12. In the photodegradation process, the recombination of photo generated electron–hole pairs has to be reduced. Here, the mixture of Fe_2_O_3_ with GO significantly prevents the rise of recombination rate on the surface of thin film. In fact, reduced GO acts as an acceptor in α-Fe_2_O_3_/GO nanocomposite layer and consequently decreases the charge recombination in the photocatalytic process. Thus, the presence of GO makes a great impact on the removal efficiency of organic dye.

**Figure 13:**
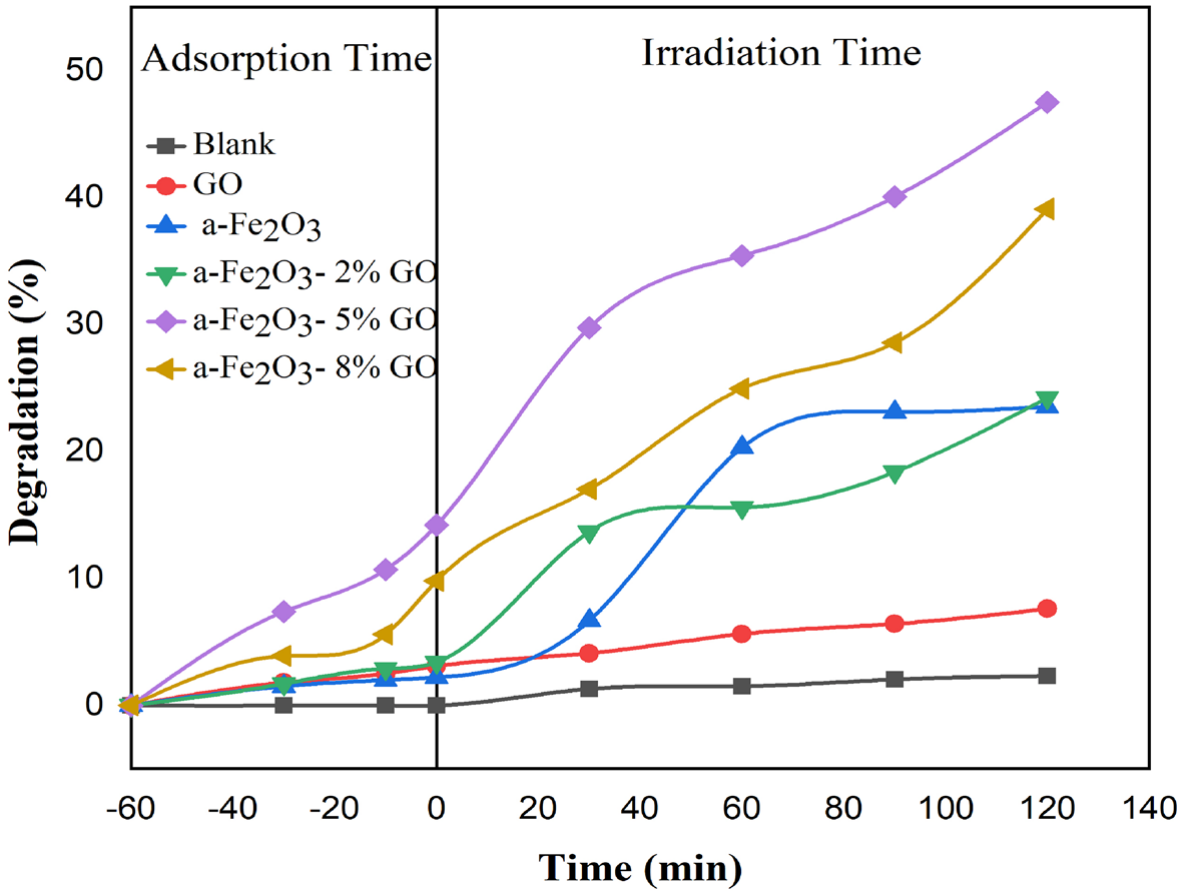
The result of photocatalytic process for all synthesized thin film layers.

In a common photocatalytic degradation system, photocatalyst releases a significant amount of electron–hole pairs in order to join in the oxidation and reduction process for degradation. Electrons react with oxygen to produce oxy radicals while holes contribute in oxidation process with hydroxyl ions to create hydroxyl radicals, which decompose pollutants in degradation process. In this study, degradation process is different from common system, which was mentioned above. Here, GO accepts photo generated electrons in their structures which significantly helps the suppression of recombination rate in the system. In this case, if GO receives enough electrons, they will share these electrons with free oxygens in the solution and therefore produce oxy radical to decompose pollutants in the system. The probable mechanism is that the excited electrons transfer from conduction band to valence band and will eventually become trapped on the surface of GO and fail to return to conduction band. On the other hand, the generated holes in conduction band of hematite produce enough hydroxyl radicals on the surface of layer to react with rhodamine molecules and finally turn them into harmless materials such as H_2_O. Figure 13 shows the mechanism of α-Fe_2_O_3_/GO thin layer under the irradiation of xenon lamps.

Although thin film α-Fe_2_O_3_/GO showed a great performance in RhB removal, the powder α-Fe_2_O_3_/GO had more removal efficiency. It seems using powder nanocomposites in the slurry system provided more photo-generated electron–hole pairs which participated in degradation reactions to decompose RhB. Despite higher recombination rate, the powder α-Fe_2_O_3_/GO contacted more with cationic dye. GO molecules covering the surface of hematite absorbed more dye molecules because they were freely in contact with all molecules in the solution. As a result, the produced electrons and holes on the surface of α-Fe_2_O_3_/GO powders decompose dye molecules more effectively. Looking at photocatalytic mechanism of powder α-Fe_2_O_3_/GO, the change of conversion and recycling between Fe(III) and Fe(II) because of free electrons caused powder nanocomposites to be more reactive. Moreover, the electrostatic interaction and π–π stacking of α-Fe_2_O_3_/GO caused α-Fe_2_O_3_/GO molecules to attract a huge number of dye molecules^43^. Consequently, the high rate of decolorization occurred as xenon lights hit the surface of nanocomposites. On the other hand, α-Fe_2_O_3_/GO also showed an acceptable removal efficiency in the photoelectrochemical reactor. The combination of reduced GO with hematite not only increased the stability of thin films for reusability process but also reduced recombination rate of generated electron–hole pairs resulting in better degradation process. However, this is an immobilized system and therefore lower amount of α-Fe_2_O_3_/GO molecules are in contact with dye molecules compared to α-Fe_2_O_3_/GO powder nanocomposites. This may lead to a considerable reduction in photodegradation efficiency for thin film layers. However, the use of both photoelectrochemical system and the presence of reduced GO prevents electron recombination, which makes it comparable with α-Fe_2_O_3_/GO in the slurry system in terms of photodegradation efficiency.

### The reusability process

**Figure 14:**
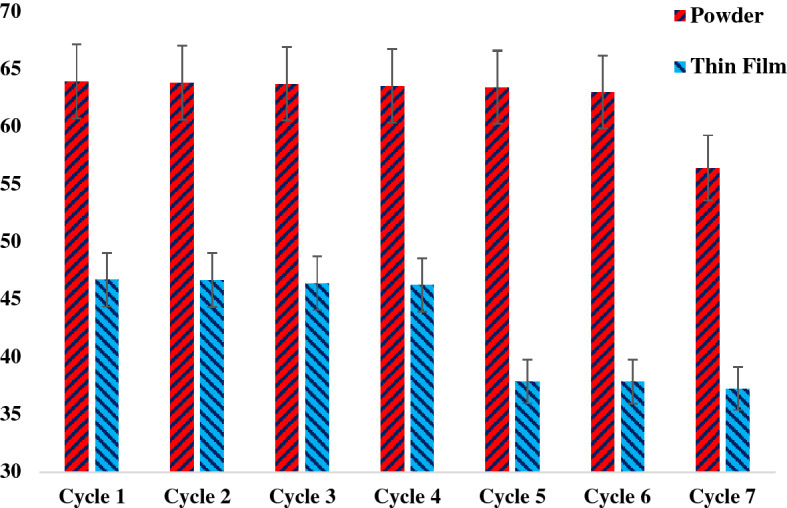
The reusability process for both thin film/FTO substrate and powder nanocomposites.

The recovery of nanocomposites with an external magnetic field was done from solution and they were tested again to measure dye removal in the next cycles. The thin-film composites were also tested in more cycles. Figure 14 shows the dye removal efficiency of each cycle for both α-Fe_2_O_3_/GO powders and thin films. α-Fe_2_O_3_/GO thin films showed acceptable performance in 7 cycles with more than 46% removal efficiency. After 4 cycles, the decline in dye removal was noticeable. A 9% decline in thin-film removal efficiency was observed due to less surface area on the surface of films to be excited and degrade dyes. In fact, dye solutions filled the space on the surface of films, leading to the reduction of photocatalytic activity. In powder nanocomposites reusability process, the α-Fe_2_O_3_/GO nanocomposites showed higher removal efficiency in more cycles compared to thin films. In fact, synthesized nanocomposites removed up to 63% of dyes in the solution in 6 cycles. This is attributed to the strong intermolecular structure of GO and hematite molecules. This strong structure increased the stability of nanocomposite and therefore removal efficiency remained high after 6 cycles. The hematite was well immobilized on the surface of GO which enhanced the surface area of nanocomposite to degrade more dye molecules. Also, the presence of GO helped the nanocomposite to adsorb more light for decomposition, which reinforce powder nanocomposites to remove dye molecules in 6 cycles with a high efficiency^48^. However, the change in removal efficiency was seen in the 7th cycle, with 7% decline in the dye removal performance. Both powder and film nanocomosites were stable in all the reuse cycles.

## Conclusions

The photocatalytic ability of thin film α-Fe_2_O_3_-GO was compared to powder α-Fe2O3-GO nanocomposites in different concentrations. Based on the results, powder nanocomposites had better performance with above 64% Rhodamine B removal efficiency from textile wastewater. The reusability process also worked better in the presence of powder nanocomposites which significantly removed dye with maximum removal efficiency in six cycles. Due to higher surface area, intermolecular connection of dyes and nanocomposite and absorption of more light, the powder nanocomposite showed better performance in photocatalytic activity under the irradiation of visible xenon lamps. On the other hand, thin film α-Fe_2_O_3_-GO displayed lower photocatalytic reaction because of less generated electron–hole pairs on the surface of layers. The characterization of both synthesized nanocomposites clearly supported the combination of hematite GO as a powder or as a thin film layer. The results show the great potential of the developed nanocomposites for high efficiency pohotocatalytic degradation of Rhodamine B and warrant further investigations into the application of these structures for the removal of other dyes and contaminants from wastewater.
